# Effect of high night temperature on storage lipids and transcriptome changes in developing seeds of oilseed rape

**DOI:** 10.1093/jxb/ery004

**Published:** 2018-02-06

**Authors:** Longhua Zhou, Tao Yan, Xin Chen, Zhilan Li, Dezhi Wu, Shuijin Hua, Lixi Jiang

**Affiliations:** 1 Institute of Crop Science, Zhejiang University, Hangzhou, China; 2 Biotech Research Institute, Shanghai Academy of Agricultural Sciences, Shanghai, China; 3 Institute of Crop and Nuclear Technology Utilization, Zhejiang Academy of Agricultural Sciences, Hangzhou, China

**Keywords:** *Brassica napus*, fatty acid composition, growth temperature at night, lipid degradation, RNA-seq analysis, seed total fatty acids

## Abstract

Global warming causes a faster increase of night temperature than of day temperature in tropical and subtropical zones. Little is known about the effect of high night temperature on storage lipids and transcriptome changes in oilseed rape. This study compared the total fatty acids and fatty acid compositions in seeds of two oilseed rape cultivars between high and low night temperatures. Their transcriptome profiles were also analyzed. High night temperature significantly affected the total fatty acids and fatty acid compositions in seeds of both low and high oil content cultivars, namely Jiuer-13 and Zheyou-50, thereby resulting in 18.9% and 13.7% total fatty acid reductions, respectively. In particular, high night temperature decreased the relative proportions of C18:0 and C18:1 but increased the proportions of C18:2 and C18:3 in both cultivars. In-depth analysis of transcriptome profiles revealed that high night temperature up-regulated gibberellin signaling during the night-time. This up-regulation was associated with the active expression of genes involved in fatty acid catabolism, such as those in β-oxidation and glyoxylate metabolism pathways. Although the effect of temperature on plant lipids has been previously examined, the present study is the first to focus on night temperature and its effect on the fatty acid composition in seeds.

## Teaser

High night temperature lowers the total fatty acids of seed and increases the proportion of 18-carbon unsaturated fatty acids by enhancing gibberellin signaling and the activity of the genes involved in fatty acid catabolism.

## Introduction

Oilseed rape (*Brassica napus* L.), an important source of edible oil worldwide, is harvested mainly for its seeds that contain a considerable amount of oil in the form of triacylglycerols (TAGs). Seed oil content (SOC) and fatty acid (FA) composition determine the quality and economic value of oilseed rape. Understanding the underlying molecular mechanism of lipid metabolism in developing seeds is a pre-condition for conducting molecular breeding toward high SOC varieties (Delourme *et al.*, 2006; Wittkop *et al.*, 2009).

Seed development is a critical process in the life cycle of a plant. In cruciferous seeds, several major transcription factor genes, namely *LEAFYCOTYLEDON1* (*LEC1*), *LEC2*, *FUSCA3*, and *ABSCISIC ACIDINSENTIVE3*, regulate numerous reactions in biochemical pathways that contribute to assimilate reservation in developing seeds (Kroj *et al.*, 2003; Mu *et al.*, 2008; Fatihi *et al.*, 2016). A variety of plant hormones affect seed development and the formation of storage compounds. Among these hormones, gibberellins (GAs) regulate seed lipid accumulation negatively by up-regulating a group of GDSL-type lipases. A part of the GDSLs that hydrolyze lipids in seeds was defined as seed fatty acid reducer (SFAR). For instance, *SFAR1*–*SFAR5* act downstream of the GA signal pathway to lower the seed lipid storage in Arabidopsis (Chen *et al.*, 2012*a*). GAs are also involved in seed germination, a process in which lipids are degraded to release energy and the carbon skeleton for establishment of young seedlings (Cao *et al.*, 2006). In addition, abscisic acid (ABA), which functions antagonistically to GA, promotes seed dormancy and inhibits seeds from germinating. The endogenous balance of GA and ABA in developing seeds determines the development toward seed germination or dormancy (Okamoto *et al.*, 2006; Nakashima *et al.*, 2009).

Seed oil content among oilseed rape cultivars varies widely from 35% to 55% depending on ecological zones and climate conditions year to year. TAGs are the main storage lipid in oilseeds, combining with oleoisin to form oil bodies with diameters of 1–2 μm in size (Chen *et al.*, 2012*b*). They can be degraded into free FAs by a variety of lipases (Li-Beisson *et al.*, 2013). The free FAs are subjected to β-oxidation, a process in which FAs are degraded into acetyl-CoA and the acetyl-CoA is subsequently converted into 4-C compounds via the glyoxylate cycle which takes place partially in the peroxisome and partially in the cytoplasm (Pracharoenwattana and Smith, 2008; Borek *et al.*, 2015). These 4-C compounds are then shipped to the mitochondria to synthesize malate, which either is used for respiration or is transported to the cytosol for gluconeogenesis (Goepfert and Poirier, 2007).

SOC is a quantitative trait that is influenced by a range of factors, among which the photosynthesis and respiration of the silique wall are essentially important. The expression of *WRINKLED1*, an important lipid synthesis regulatory gene in developing seeds, is associated with silique wall photosynthetic activity (Weselake *et al.*, 2009; Hua *et al.*, 2012). Light intensity, daytime temperature (DT), night temperature (NT), and carbon dioxide concentration are essential factors in determining the efficiency of photosynthesis. Plants adapt their daily growth to the thermo periodic cycle by the evolution of thermo-period responses. An appropriate DT is necessary for plant growth and the biosynthesis of seed storage compounds-. However, a DT that exceeds a certain threshold reduces the SOC by repressing photosynthesis genes (Zhu *et al.*, 2012).

Temperature is one of the most important environmental factors determining plant distribution. Drastic changes in temperature result in plant adaptation with modified polyunsaturated FA (PUFA) concentrations in their membranes and storage lipids. PUFAs in seed lipids are catalyzed by fatty acid desaturases (FADs), particularly FAD2 that converts oleic acid (C18:1) to linoleic acid (C18:2) and FAD3 that catalyzes conversion of linoleic acid (C18:2) to linolenic acid (C18:3). Temperature can affect the degree of FA desaturation indirectly through its effects on substrate availability (Li *et al.*, 2015; Menard *et al.*, 2017). Temperature can also directly influence *FAD2* and *FAD3* expression to various degrees, depending on species, tissues, and gene (Tang *et al.*, 2005; O’Quin *et al.*, 2010). Previous studies reported a significant up-regulation of *FAD2* and *FAD3* at low temperature (Román *et al.*, 2012; Zhu *et al.*, 2012). Other reports indicated either down-regulation or no significant change in the expression levels of *FAD2* and *FAD3* at low temperature (Heppard *et al.*, 1996; Li *et al.*, 2015). 18-C fatty acids, such as oleic acid (C18:1) and linoleic acid (C18:2), are dominant FA forms in seeds of most oilseed rape cultivars nowadays.

Global warming causes a faster increase of NT than of DT in tropical and subtropical zones; this increase is unfavorable for the accumulation of assimilates in crop storage organs (Iyer *et al.*, 2008; Lesk *et al.*, 2016). Nevertheless, little is known about the effect of high NT on total FAs and FA compositions in oilseed rape and how developing seeds respond to high NT with transcriptome changes. In the present study, we compared the total FAs, FA compositions, and transcriptomes of developing seeds in two oilseed rape cultivars, namely Jiuer-13 (JR) and Zheyou-50 (ZY), between high and low NT conditions. This study aimed to determine the significant transcriptome changes resulting from high NT treatment and a mechanism interfering with lipid catabolism in seeds during the night.

## Materials and methods

### Plants materials and growth conditions

**Fig. 1.**
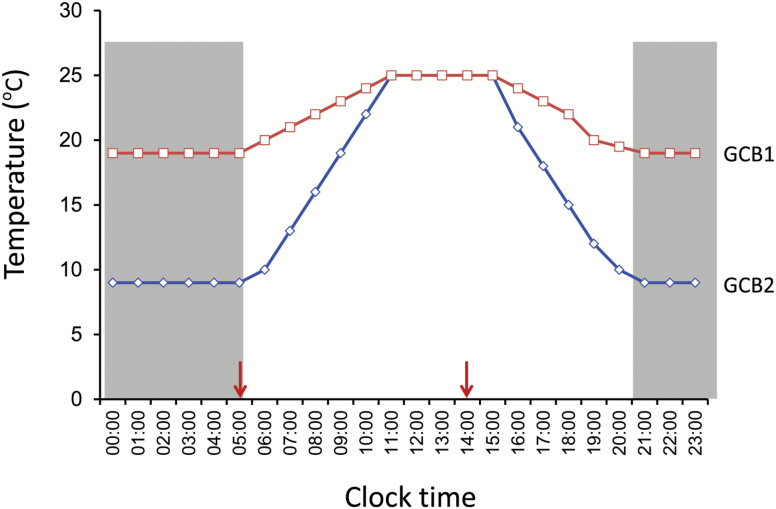
Design of the temperature treatment experiment in growth chambers. Plants were moved to growth chambers after the opening of the first flower. Different daily temperature cycles were set in two growth chambers, namely growth chamber 1 (GCB1) and GCB2. Curves indicate the daily temperature changes in the low (diamonds) and high (squares) NT chambers. The gray shaded area shows the time zone between 21.00 h and 05.00 h when the light was off in the chambers. Light in the chambers was on during 05.00–21.00 h. The arrows point to the time when developing seeds for RNA extraction were collected.

Two oilseed rape (*Brassica napus* L.) cultivars, namely Zheyou-50 (ZY) and Jiuer-13 (JR), were used in the study. ZY has a relatively high SOC (50%) and nearly zero erucic acid (EA) (C22:1), whereas JR is an old local cultivar which had an SOC of ~35% and an EA proportion >30%. Seeds were germinated in pots and the seedlings were grown on nutrient soil (Shengsheng Co., Ltd, Guangzhou, China). On the day when the first flowers bloomed, two plants of similar size from each cultivar were moved to two separate growth chambers (GCBs) (Zeda Instrument Co., Ltd, Hangzhou, China), namely GCB1 and GCB2. As illustrated in Fig. 1, the daily light/dark cycle (light from 05.00 h to 21.00 h and darkness from 21.00 h to 05.00 h) and the daytime light intensity, which was 306 μmol light quantum m^−2^ s^−1^, were controlled in exactly the same way for both GCB1 and GCB2. As illustrated, the thermo cycle for GCB1 was 9 °C between 21.00 h and 05.00 h, increased from 9 °C to 25 °C between 05.00 h and 11.00 h, was maintained at 25 °C between 11.00 h and 15.00 h, and decreased from 25 °C to 9 °C between 15.00 h and 21.00 h. The DT between 11.00 h and 15.00 h in GCB2 was the same as that in GCB1; however, its NT between 21.00 h and 05.00 h was 19 °C, 10 °C higher than that in GCB1. The slope of temperature increase/decrease in GCB1 between 05.00 h and 11.00 h/15. 00 and 21.00 h was flatter than that in GCB2 (Fig. 1). Altogether, eight treatments A–H, which were a combination of two thermo cycles, two cultivars, and two harvest times of the developing seed, are defined in Table 1. Individual flowers were tagged with flowering dates. The 16 DAP (days after pollination) seeds, in which lipid biosynthesis was active, were harvested and frozen in liquid nitrogen immediately after detachment. The seeds were used for RNA extraction, the consequent RNA sequencing (RNA-seq) experiments, and analysis of endogenous GA levels.

**Table 1.** RNA-seq analysis codes for the eight treatments combining the factors such as NT, genotype, and sample harvesting time

Codes	Genotype	NT treatment	Harvest time
A	JR	High NT	14.00 h
B	JR	High NT	05.00 h
C	JR	Low NT	14.00 h
D	JR	Low NT	05.00 h
E	ZY	High NT	14.00 h
F	ZY	High NT	05.00 h
G	ZY	Low NT	14.00 h
H	ZY	Low NT	05.00 h

### Assay of total FAs and FA composition

Mature seeds of ~50 DAP were harvested and air-dried until their weight remained constant for the assay of total FAs and FA composition using a gas chromatograph (SHIMADZU GC-2014, Kyoto, Japan,). Three biological repeats of each treatment were taken for the assay. The procedures for FA extraction and conditions for GC analysis were according to our previous description (Zhu *et al.*, 2012; Chen *et al.*, 2012a, b; Wang *et al.*, 2014). Total FAs were calculated by adding up the 11 FA species, namely myristic acid (C14:0), palmitic acid (C16:0), stearic acid (C18:0), oleic acid (C18:1), linoleic acid (C18:2), linolenic acid (C18:3), arachidic acid (C20:0), eicosenoic acid (C20:1), docosanoic acid (C22:0), erucic acid (C22:1), and tetracosanoic acid (C24:0), detected by GC with internal standards. Data were classified with Win-Excel and analyzed via one-way ANOVA using the statistical package SAS (Version 9.0, SAS Institute, Inc.). Comparisons between the means of each treatment were made by Duncan’s multiple range test at a level of *P*≤0.05 for a significant difference.

### RNA extraction and RNA-seq experiment

Total RNAs were isolated from 16 DAP seeds using Trizol according to the manufacturer’s instructions (Invitrogen, Carlsbad, USA). Sequencing libraries were prepared following the IIIumina RNA Seq library kit guide (New England BioLabs Inc., Ipswich, USA) only with minor modifications. Total RNAs digested by DNase I were enriched using oligo(dT) magnetic beads and a fragmentation reagent was added (inside the library prep kit) in a Thermomix (inside the library prep kit). The first and second cDNA strands were synthesized using the fragmented mRNA as template and enriched by magnetic beads AgencourtAMPure XP (Beckman Coulter, Shanghai, China). They were repaired at the ends, and A-tails were added at the 3' end. Fragments that were 300–350 bp in size were selected through a nucleic acid extractor of the EASYspin Plus Plant RNA Kit (Gene-Foci Biotech, Beijing, China) and were enriched by 15 cycles of PCR and purified on an agarose gel. The amplified libraries were checked by an Agilent 2100 Bioanalyzer (Invitrogen) and an ABI StepOnePlus Real-Time PCR System (ThermoFisher, Shanghai, China) subsequently, and were sequenced by the Illumina HiSeq™ 2500 (Illumina, San Diego, USA). Quality control was performed by deleting the reads that contained >5% N.

### Blasting clean reads to the reference genome

Clean RNA-seq reads were mapped against the reference genome assemblies of oilseed rape (*B. napus.* L) (Chalhoub *et al.*, 2014) using SOAPaligner/SOAP2 software. The gene expression level was quantified using the same reference genome. The quality control of the nucleotide mismatches was less than five bases. Gene expression levels represented by read numbers were normalized to relative abundance as reads per kilobase of transcript per million mapped reads (RPKM) (Mortazavi *et al.*, 2008). Differentially expressed genes (DEGs) between treatments were screened using the R package DESeq2 (Love *et al.*, 2014). The threshold value was set by |log_2_ratio|≥1 and false discovery rate (FDR)≦0.01.

### K-means clustering

K-means clustering was performed by the ‘kmeans’ function in R package ‘stats’ (v. 3.2.2), where K value refers to the number of clusters grouped in the data set. The genes used here is the union of DEGs. The algorithm randomly assigned each gene into one of nine clusters and located the centroid of each cluster according to the average RPKM value in two replicates. The data were normalized as log_2_^(RPKM+1)^. The following steps were iterated until variation within the cluster could not be reduced any further: (i) reassign data points to the cluster whose centroid is closest; and (ii) calculate the new centroid of each cluster. Within-cluster variation was calculated as the sum of the Euclidean distance between the data points and their respective cluster centroids.

### KEGG enrichment

The metabolic or signal transduction pathways where the genes might be involved were assigned by blast against the KEGG (Kyoto Encyclopedia of Genes and Genomes) database, with an E-value cut-off of 1E-05. The significantly enriched pathways associated with DEGs were identified using the whole expressed genes as background. Enrichment analysis was performed by the software path-finder (in-house designed by 1Gene Technology Company) with a *q*-value 0.05 as the confidence level. The degree of KEGG enrichment is measured by the rich factor, *q*-value, and the number of genes enriched in the pathway. The rich factor refers to the ratio of the number of DEGs in the pathway to the number of all genes annotated (Mortazavi *et al.*, 2008; Love *et al.*, 2014).

### Multiple EM for Motif Elicitation (MEME) analysis for promoter sequence

The promoter sequences of the candidate genes encoding enzymes of the glyoxylate pathway were referred to the reference genome of *B. napus* (Chalhoub *et al.*, 2014). The 1000 bp nucleotide sequence upstream of the start codon of each candidate gene was downloaded and scanned for conservative sequences using the MEME method (http://meme-suite.org/). According to the method, the minimum and maximum widths of each motif should be between 6 and 20 nucleotides. Parameters were designed such that the number of motifs was three, and the minimum and maximum width of each motif was 6 and 20 nucleotides, respectively.

### Real-time quantitative PCR (RT-qPCR) analysis

RT-qPCR experiments were performed following Udvardi *et al.* (2008). Candidate genes in β-oxidation and GA synthesis pathways were selected for RT-qPCR verification. The specific primer pairs were designed to cover a 100–200 bp region (Supplementary Table S21 at *JXB* online). The first-strand cDNAs for RT-qPCR were synthesized in a 20 μl solution (containing ~1 μg of RNA as template) using the PrimeScript™ 1st Strand cDNA Synthesis Kit following the manufacturer’s instructions (catalog no. D6110A, Takara, Japan). *BnACTIN7* was chosen as the endogenous control for standardization (Zhu *et al.*, 2012). The RT-qPCR experiments were performed using th eSYBR *Premix ExTaq*™ Kit (catalog no. RR420A, Takara) in a C1000™ thermal cycler (Bio-Rad, Shanghai, China) following the manufacturers’ instructions. The results of the comparisons were presented along with the heat map (http://bar.utoronto.ca/ntools/cgi-bin/ntools_heatmapper_plus.cgi). Parameters were designed such that the color schemes for negative, zero, and positive values were green, black, and red, respectively, whereas other parameters were default.

### Analysis of endogenous GA levels in developing seeds

The preparation of seed samples and the setting of HPLC conditions were as described by Zanewich and Rood (1993). In brief, the separation was performed on a Waters XBridge C18 column (250 nm×4.6 nm, 5 μm). Head pressure of the carrier gas (He) was 265 kPa, resulting in a flow speed of ~1.4 ml min^–1^. The temperature program was set as follows: a rapid temperature ramp of 25 °C min^–1^, to 200 °C, a decreased rate of temperature ramp of 5 °C to 270 °C, and a final rapid temperature ramp of 20 °C min^–1^ to 300 °C. A 10 ng aliquot of GA_1_ (Simalab, Tianjin, China) internal standard was added during the extraction. The amount of endogenous GA_1_ was calculated in light of the peak area ratios.

## Results

### Effect of NT on total FAs and FA compositions

**Fig. 2.**
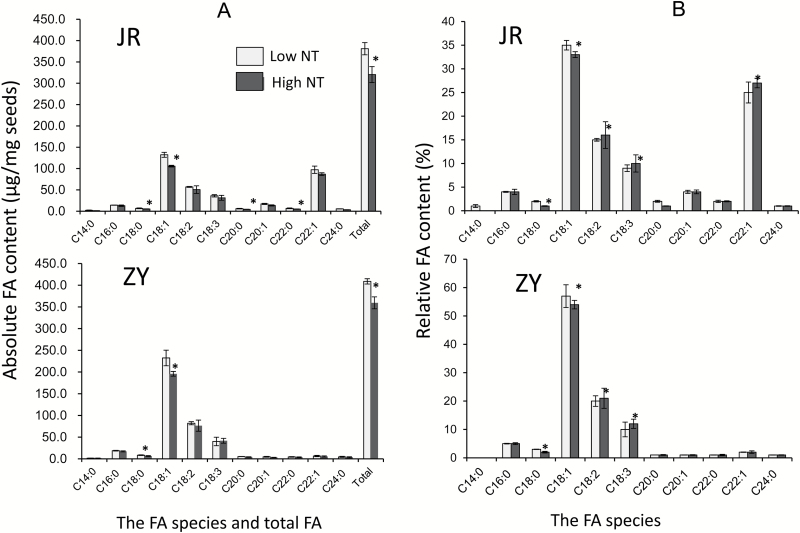
Comparisons of total FAs and FA compositions between low and high NT treatments. (A) Absolute values based on the means of biological repetitions showing total FA contents and various FA species. (B) Relative percentages of FAs in seed samples treated with low and high NTs. Asterisk indicates significant difference at *P*≤0.05 levels between the low and high NT treatments by *t*-test.

The effects of NT on the total FAs of seed and the composition of FA species, namely myristic acid (C14:0), palmitic acid (C16:0), stearic acid (C18:0), oleic acid (C18:1), linoleic acid (C18:2), linolenic acid (C18:3), arachidic acid (C20:0), eicosenoic acid (C20:1), docosanoic acid (C22:0), erucic acid (C22:1), and tetracosanoic acid (C24:0), were investigated. As shown in Fig. 2A, high NT caused low total FAs in both cultivars. Approximately 1 mg of JR and ZY seeds only contained 320.4 (±18.7) μg and 359.5 (±13.7) μg of FAs under high NT condition, which were reduced by 18.9% and 13.7% relative to the total FAs achieved under the low NT condition, respectively. In particular, high NT resulted in significantly lower C18:0, C18:1, C20:1, and C22:0 in JR seeds and significantly lower C18:0 and C18:1 in ZY seeds than those of low NT. The significant reduction of C18:0 and C18:1 and the non-significant decrease in the other FAs altered the various FA proportions in seeds (Fig. 2B). High NT decreased the relative proportions of C18:0 and C18:1 but increased those of C18:2 and C18:3 in both cultivars. Notably, in JR seeds, high NT did not result in a significant reduction in the absolute content of C22:1, although C22:1 accounted for the second largest group of individual FA species (Fig. 2A); instead, high NT increased its relative proportion (Fig. 2B).

### Effect of NT on transcriptome changes

#### Quality of RNA-seq experiments

To explore the changes in seed transcriptome in response to high NT, we performed an RNA-seq experiment. The quality of the RNA-seq experiment depends on the read coverage with respect to the reference genome of oilseed rape and the correlations between biological repetitions. As listed in Supplementary Table S1, an average of 63.9% of all reads from 16 independent RNA samples (two repetitions for each treatment from A to H as defined in Table 1) showed >80% nucleotide similarities to the reference genome (Chalhoub *et al.*, 2014). Moreover, the correlations between two repetitions for each treatment were >0.90. These data provided the basis for further in-depth transcriptome profile analysis.

#### Overall number of expressed genes and range of transcriptional levels

**Fig. 3.**
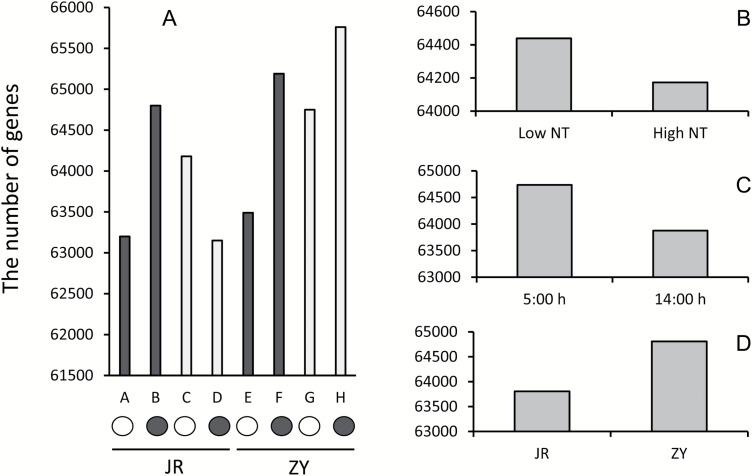
Overall number of expressed genes detected and gene expression level. (A) Comparison of the expressed gene numbers between eight treatments (A–H), (B) low and high NT treatments, (C) different time points when samples were harvested, and (D) two cultivars. The letters A, B, E, F represent low NT and C, D, G, H represent high NT on the X-axis in (A) represent high and low NT treatments, respectively. The open and filled circles) in (A) indicate the harvest time at 05.00 h and 14.00 h, respectively. Values are denoted in means of two repetitions in (A) and four treatments with two repetitions each in (B–D).

The total number of genes expressed in each treatment ranged from 63061 to 65788 according to the threshold set (Fig. 3A). On average, the samples treated with high NT displayed 266 less expressed genes than those treated with low NT (Fig. 3B). Samples harvested at 14.00 h in the daytime showed 861 less expressed genes than those collected at 05.00 h in darkness (Fig. 3C). Samples from cultivar JR exhibited 1002 less expressed genes than those from cultivar ZY (Supplementary Fig. S1). The gene expression levels, which were normalized with RPKM (log_10_^X^), demonstrated a similar range of transcript levels in all eight treatments (A–H) (Table 1; Supplementary Fig. S1).

#### DEGs caused by different NTs

**Fig. 4.**
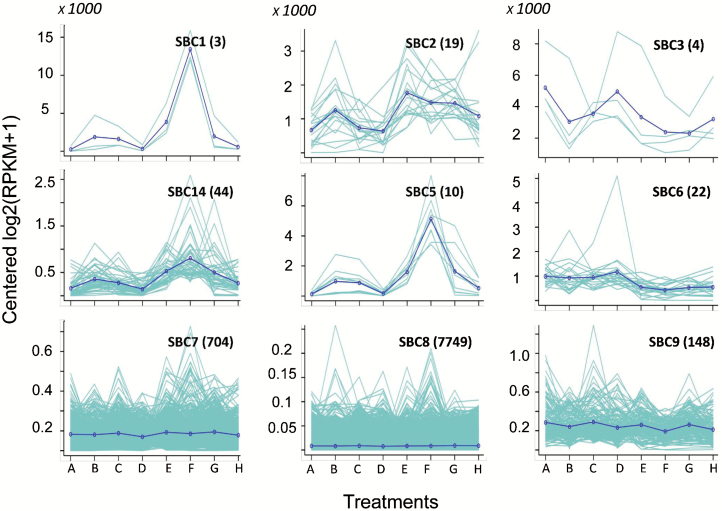
K-means cluster analysis for transcriptional changes in the oilseed rape genome against treatments A–H defined in Table 1. Nine groups of genes were classified as subcluster 1 (SBC1) to SBC9. The number of genes in each SBC is indicated in parentheses. The scales on the *y*-axis should be magnified 1000 times.

**Fig. 5.**
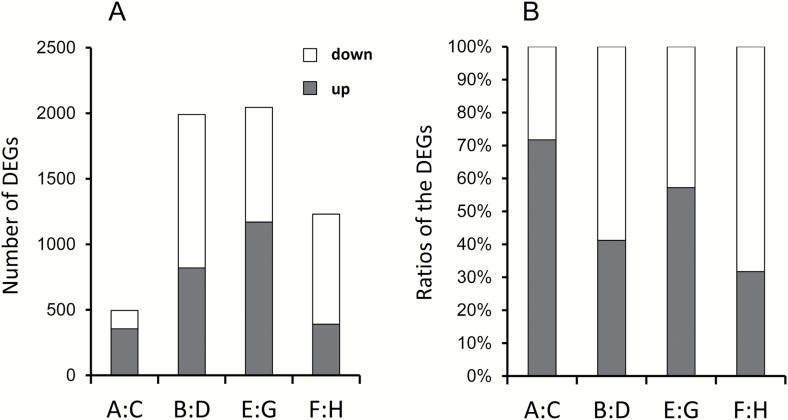
Pairwise comparisons of the number of DEGs and ratios of DEGs between the low and high NT treatments (A) Number of DEGs. (B) Ratios of up- and down-regulated genes. White and dark gray bars indicate lower and higher gene expression in samples treated with high NT relative to those treated with low NT, respectively. A–H represent the eight treatments defined in Table 1. Up-regulation (or down-regulation) is defined as the log_2_^X^ ≥1 (or log_2_^X^ ≤1), where X equals the ratio of the transcriptional level of genes in samples treated with high NT divided by those treated with low NT.

To understand the NT effect, we compared the DEGs between high and low NT treatments. In brief, paired samples A and C, E and G, B and D, and F and H were different from each other only because of NT. Up-regulation (or down-regulation) was defined as the log_2_^X^ ≥1 (or log_2_^X^ ≤ −1), where X equals the ratio value of the transcriptional level of genes in samples treated with high NT divided by those treated with low NT. K-means cluster analysis was applied to classify the DEGs in the allotetraploid genome of oilseed rape. Nine clusters were classified on the basis of expressional changes against the eight treatments (A–H) (Table 1; Fig. 4). Genes exhibited significant differences in expression between NT treatments in clusters SBC1, SBC2, SBC3, SBC4, SBC5, and SBC6 (listed in Supplementary Tables S2–S7). Additionally, the total number of DEGs caused by different NTs was compared between A and C, B and D, E and G, and F and H (Fig. 5A). Results showed higher amounts of DEGs in ZY than in JR. High NT caused more up-regulations in samples harvested at 14.00 h (A, C, E, and G) than that at 05.00 h (B, D, F, and H) (Fig. 5B). DEG IDs between A and C, B and D, E and G, and F and H are provided in Supplementary Tables S8–S11. We further performed KEGG analysis to identify the DEGs caused by NT. The top 20 pathways for DEGs between A and C, B and D, E and G, and F and H with numbers of DEGs and rich factors are illustrated in Supplementary Figs S2–S5; the corresponding gene IDs are provided in Supplementary Tables S12–S15. KEGG analysis revealed that in samples harvested at 14.00 h during the daytime, the highest magnitude of changes caused by NT differences was observed in starch metabolism, pentose phosphate, and carbon fixation pathways in photosynthetic organisms. Nonetheless, in samples harvested at 05.00 h in darkness (B/D, E/G), the highest magnitude of changes resulting from NT differences was found in glyoxylate and dicarboxylate metabolism and glycolysis/gluconeogenesis, which were involved in FA catabolism (Supplementary Figs S2–S5).

#### DEGs in pathways involved in and/or related to FA metabolism caused by different NTs

To understand the NT effect on FA metabolism, we focused on the DEGs in pathways that are remarkably directly involved in and/or related to FA metabolism. The numbers of up- and down-regulated DEGs caused by high NT are listed in Table 2; their corresponding IDs are provided in Supplementary Tables S16–S19. Pairwise comparisons were performed between the treatments A and C, B and D, E and G, and F and H. A total of 22 up-regulated genes and 65 down-regulated genes were detected in sample A relative to those in sample C, and a total of 140 up-regulated and 240 down-regulated genes were detected in sample E relative to those in sample G. These results showed that high NT caused less up-regulated genes than down-regulated genes in the samples harvested at 14.00 h in the daytime. Additionally, 254 genes were up-regulated and 150 were down-regulated in sample B relative to those of sample D, and 192 genes were up-regulated and 76 were down-regulated in samples E and G relative to samples F and H. These results indicated that high NT caused more up-regulated genes than down-regulated genes in samples harvested at 05.00 h in darkness. High NT caused the highest magnitude of changes in pathways, such as plant hormone signaling, starch metabolism, and protein processing in the endoplasmic reticulum, which together accounted for >35% of the total DEGs found in 22 pathways. For DEGs in cutin biosynthesis, FA elongation, and oxidative phosphorylation pathways, high NT caused more down-regulation than up-regulation in samples collected at 14.00 h and at 05.00 h. For the DEGs in FA biosynthesis, unsaturated FA biosynthesis, pyruvate metabolism, and citrate cycle pathways, high NT resulted in more up-regulation than down-regulation in samples collected at 14.00 h and at 05.00 h. Moreover, for genes in plant hormone signaling, citrate cycle, and glyoxylate metabolism pathways, high NT resulted in a high number of up-regulated genes in samples harvested at 05.00 h in darkness regardless of the genotype. High NT treatments caused an evident genotypic difference between the amount of DEGs in JR and ZY. A higher number of DEGs was observed in JR than in ZY among samples harvested at 05.00 h. In contrast, more DEGs were observed in ZY than in JR among samples harvested at 14.00 h (Table 2).

**Table 2.** DEGs enriched on the pathways involving FA metabolism

Pathways	A/C		B/D		E/G		F/H	
	≥1	≦ –1	≥1	≦-1	≥1	≦ –1	≥1	≦ –1
Circadian rhythm	0	3	6	4	9	5	8	3
Carbon fixation	0	4	22	6	6	8	10	2
Photosynthesis	0	1	25	0	6	4	6	0
Cutin biosynthesis	0	0	0	5	5	9	4	5
Glycerolipid metabolism	0	1	4	5	4	9	3	2
Glycerophospholipid metabolism	4	1	2	3	3	12	4	3
Fatty acid biosynthesis	0	0	10	0	7	5	12	1
Fatty acid elongation	0	2	1	7	1	4	0	2
Fatty acid metabolism	0	1	2	4	2	2	3	0
Unsaturated FA biosynthesis	0	0	14	0	4	3	14	0
α-Linolenic acid	2	0	3	7	5	6	6	7
Protein processing in ER	1	17	16	19	11	30	5	4
Glyoxylate metabolism	0	1	15	2	6	5	5	2
Glycolysis/gluconeogenesis	1	2	20	7	8	11	14	1
Starch metabolism	6	12	43	25	23	24	34	21
Pyruvate metabolism	1	1	10	4	8	5	9	3
Citrate cycle (TCA cycle)	1	0	4	4	3	2	3	1
Oxidative phosphorylation	1	3	5	5	6	13	2	3
Pentose phosphate pathway	0	4	9	3	3	6	5	0
Brassinosteroid biosynthesis	1	0	2	6	2	4	3	1
Indole alkaloid biosynthesis	0	1	3	6	2	3	7	2
Plant hormone signal	4	11	38	28	16	38	35	13
**Total**	**22**	**65**	**254**	**150**	**140**	**204**	**192**	**76**

Treatments A–H are defined as in Table 1.

We showed that high NT resulted in a higher number of up-regulated genes than down-regulated genes in carbon fixation and starch metabolism pathways in both JR and ZY samples harvested at 05.00 h. However, high NT caused a higher number of down-regulated genes than up-regulated genes in carbon fixation and starch metabolism pathways in both JR and ZY samples harvested at 14.00 h (Table 2). The result demonstrated a positive effect of high NT on the expression of genes in carbon fixation and starch/sucrose metabolism at 05.00 h in darkness. In contrast, a negative effect was observed in samples harvested at 14.00 h during the daytime.

#### DEGs in pathways of plant hormone signaling and glyoxylate metabolism caused by different NTs

**Fig. 6.**
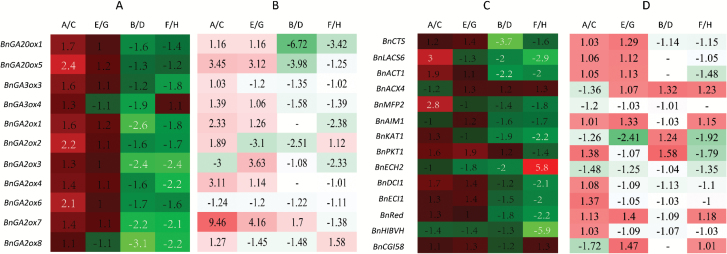
Comparison between RT-qPCR and RNA-seq results for DEGs caused by different NTs. (A and C) Results from RT-qPCR. (B and D) Results from RNA-seq. (A) and (B) are DEGs on the GA synthesis pathway, and (C) and (D) are DEGs on the β-oxidation pathway. The numbers are log_2_^X^-normalized ratio values corresponding to the transcriptional level of genes in samples treated with high NT divided by those treated with low NT. Red color represents higher gene expression levels of those treated with high NT than those treated with low NT. Green color corresponds to lower gene expression levels of those treated with high NT than those treated with low NT. The darkness of the red or green color represents the absolute value of up- or down-regulation by high NT. The blocks without a numerical value indicate that the gene expression was not detected by RNA-seq.

**Fig. 7.**
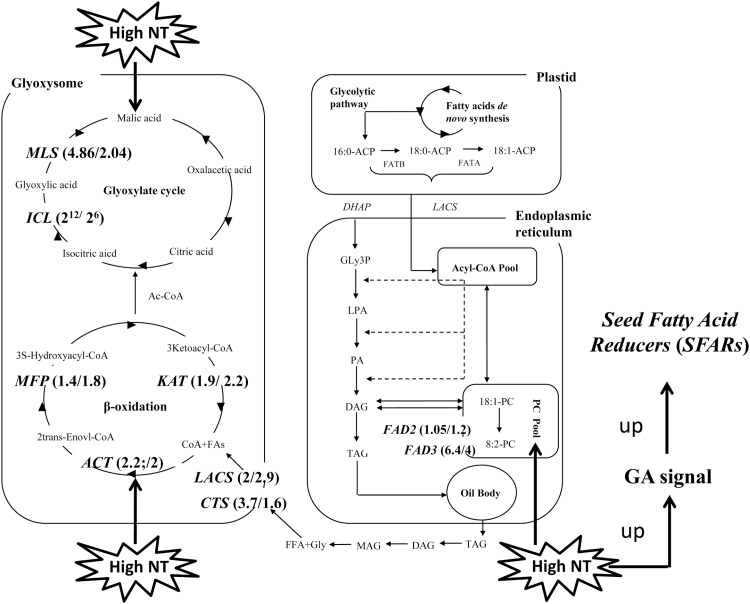
Schematic representation of the influence of high NT on FA anabolism and catabolism biosynthetic pathways. High NT promoted the expression of the genes in the FA biosynthesis pathway (Table 2; Supplementary Table S17), in particular *FAD2* and *FAD3*, but also the genes enhancing GA signal (Fig. 6). The enhanced GA signal resulted in the increased expression of *SFAR* genes (Supplementary Table S9) (right). The free FAs are subjected to β-oxidation, a process in which FAs are degraded into acetyl-CoA. Subsequently, acetyl-CoA is converted into 4-C compounds via the glyoxylate cycle, which occurs partially in the peroxisome (left). The numbers in parentheses indicate the up-regulated folds of the genes in relevant cycles. *FAD*, fatty acid desaturase; *MLS*, malate synthase; *ICL*, isocitrate lyase; *MFP*, multifunctional protein; *KAT*, 3-keto-acyl-CoA thiolase; *ACT*, anthocyanin 5-aromatic acyltransferase; *ICL*, isocitrate lyase; and cts, comatose.

Transcriptome profile analysis discovered a considerable number of DEGs in plant hormone pathways involving GA signaling (Table 3). Notably, genes involved in GA biosynthesis, such as *BnaA09g01090D* (*BnGA1*), *BnaA06g27960D* (*BnGA3*), *BnaC07g28980D* (*BnGA3*), and *BnaC05g11920D* (*BnGA4*), were up-regulated in the samples harvested at 05.00 h. In contrast, DELLA genes were down-regulated, thereby indicating a strong GA signaling caused by high NT. These genes included *BnaCnng68300D* (*BnRGL1*), *BnaA07g01720D*, *BnaA07g01720D* (*BnRGL1*), *BnaC05g47760D* (*BnRGL3*), and *BnaC07g20900D* (*BnRGA*), which functioned as negative regulators in GA signaling pathways. The most remarkably changed DEG was a GA negative regulator, namely *BnaCnng68300D*, of which high NT caused a >82.5-fold down-regulation after log_2_^X^ normalization. To investigate whether high NT caused a higher GA level in developing seeds harvested at 05.00 h, we compared the endogenous GA_1_ concentrations between the high NT (B, F) and low NT (D, H) treatments, since GA_1_ and its precursors are the principal bioactive GAs in Brassica seeds (Zanewich and Rood, 1993). A 1 mg aliquot of the seed tissues of the samples contained ~0.35 ng of GA_1_. There were no significant differences in the GA_1_ level between the high and low NT treatments. On the other hand, genes involved in glyoxylate metabolism and β-oxidation were up-regulated in samples B and F, which showed the active reactions of lipid catabolism (Table 4; Figs 6, 7).

**Table 3.** DEGs caused by different NTs in pathways involving GA signaling in samples harvested at 05.00 h in the dark

Gene ID	Detected in genotype	Arabidopsis orthologs	Up- or down-regulation caused by high NT	Annotation of gene function
*BnaA09g01090D*	JR and ZY	*GA1*	Up (1.8/1.36)	GA biosynthesis
*BnaA09g26500D*	JR	*GA2OX2*	Up (2.28*)	GA biosynthesis
*BnaC07g09260D*	JR and ZY	*GA2OX2*	Up (2.46*/1.03)	GA biosynthesis
*BnaA05g17600D*	ZY	*GA2OX4*	Up (1.37)	GA biosynthesis
*BnaA06g27960D*	JR and ZY	*GA3*	Up (1.62/1.36)	GA biosynthesis
*BnaC07g28980D*	JR and ZY	*GA3*	Up (1.92/1.28)	GA biosynthesis
*BnaC05g11920D*	JR	*GA4*	Up (1.74)	GA biosynthesis
*BnaC01g17380D*	JR	*GA5*	Up (4.2*)	GA biosynthesis
*BnaA03g47400D*	JR and ZY	*GA5*	Up (7.52*/1.23)	GA biosynthesis
*BnaC07g39650D*	JR and ZY	*GA5*	Up (6.62*/4.06*)	GA biosynthesis
*BnaCnng68300D*	ZY	*RGL1*	Down (–82.3*)	GA negative regulator
*BnaA07g01720D*	JR	None	Down (–2.7*)	GA negative regulator
*BnaCnng28010D*	JR and ZY	*RGL1*	Down (–1.46/–2.06*)	GA negative regulator
*BnaC05g47760D*	JR and ZY	*RGL2*	Up (1.7/1.75)	GA negative regulator
*BnaC09g40420D*	JR and ZY	*RGL3*	Down (–1.06)/Up (1.4)	GA negative regulator
*BnaC07g20900D*	ZY	*RGA*	Down (–1.21)	GA negative regulator
*BnaC06g02750D*	JR	*GID1*	Down (–2.34*)	Response to GA
*BnaA06g02980D*	JR	*GID1*	Down (–2.36*)	Response to GA
*BnaA02g33270D*	JR	*GID1*	Down (–2.03*)	Response to GA

In the fourth column, the asterisks indicate >4-fold up- or down-regulation caused by high NT, and the slashes separate the data for JR and ZY.

**Table 4.** DEGs caused by different NTs in pathways involving β-oxidation and glyoxylate metabolism

Gene ID	Detected in genotype	Arabidopsis orthologs	Up-regulation caused by high NT	Annotation of gene function
*BnaA08g16170D*	JR and ZY	*CTS*	1.17/1.15	Fatty acid β-oxidation
*BnaC03g60720D*	JR and ZY	*CTS*	1.12/1.37	Fatty acid β-oxidation
*BnaA09g32380D*	JR	*ACX4*	1.39	Fatty acid β-oxidation
*BnaA04g05950D*	JR	*ACX4*	1.17	Fatty acid β-oxidation
*BnaA01g07910D*	ZY	*AIM1*	1.36	Fatty acid β-oxidation
*BnaA02g00100D*	JR and ZY	*PMDH2*	2.21*/3.87*	Fatty acid β-oxidation
*BnaA06g08600D*	JR	*PMDH2*	2.26*	Fatty acid β-oxidation
***BnaA03g36420D***	**JR and ZY**	***ICL***	**2** ^**12**^ */2 ^**6**^ *	**Rate-limiting in glyoxylate cycle**
***BnaC02g02870D***	**ZY**	***MLS***	6.92*	**Rate-limiting in glyoxylate cycle**
*BnaC03g38540D*	JR and ZY	*GOX1*	4.13*/3.39*	Glyoxylate cycle
BnaA05g25050D	JR and ZY	*GOX1*	1.73/1.86	Glyoxylate cycle
BnaC05g39220D	JR and ZY	*GOX1*	1.73/1.05	Glyoxylate cycle
*BnaA05g23020D*	JR and ZY	*ACN1*	1.06/1.23	Glyoxylate cycle
*BnaC05g36470D*	ZY	*ACN1*	1.32	Glyoxylate cycle
*BnaC08g29480D*	JR and ZY	*CSY2*	4.86*/2.04*	Glyoxylate cycle
*BnaA04g01840D*	JR and ZY	*CSY2*	1.93/1.11	Glyoxylate cycle
*BnaC04g22440D*	JR and ZY	*CSY5*	1.19/1.05	Glyoxylate cycle

In the fourth column, the asterisks indicate>4-fold up-regulation caused by high NT, and the slashes separate the data for JR and ZY.

The rows in bold show the rate-limiting enzymes in glyoxylate metabolism pathways.

*CTS*, *COMATOSE*; *ACX4*, *ACYL-COA OXIDASE 4*; *AIM1*, *ABNORMAL INFLORESCENCE MERISTEM1*; *PMDH2*, *PEROXISOMAL NAD-MALATE DEHYDROGENASE 2*; *ICL*, *ISOCITRATE LYASE*; *MLS*, *MALATE SYNTHASE*; *GOX1*, *GLYCOLATE OXIDASE 1*; *ACN1*, *ACETATE NON-UTILIZING 1*; *CSY*, *CITRATE SYNTHASE*.

#### RT-qPCR confirmation of RNA-seq results

To confirm the RNA-seq results, several genes found in GA biosynthesis and glyoxylate metabolism were selected for RT-qPCR. As shown in Fig. 6, RT-qPCR results generally matched well with the RNA-seq analysis results. High NT resulted in high expression of genes in the samples harvested at 05.00 h and low expression of genes in the samples harvested at 14.00 h in both cultivars. Only a few exceptions existed, as shown in Fig. 6.

## Discussion

In this study, we investigated the effect of high NT on total FAs and FA compositions of oilseed rape and the transcriptome changes in the developing seeds responding to high NT. Two oilseed rape cultivars, namely JR and ZY, were used in the experiments to (i) increase the reliability of the experimental results and (ii) understand the genotypic difference. Although the two cultivars demonstrated the same tendency in responding to high NT, evident genotypic differences were observed with regard to total FAs, FA composition changes, and transcriptome alterations (Figs 2–6; Tables 2–4). A notable difference between JR and ZY was the number of expressed genes under low NT conditions in samples collected at 05.00 h in darkness (Fig. 3A). A total of 2558 less expressed genes were observed in JR than in ZY (C versus G). This difference was attributed to the genotype×environment effect between NT and genotype. Analysis of the 2558 DEGs between JR and ZY revealed that a large proportion of the DEGs were involved in various metabolic pathways, partly explaining the phenotypical variations between JR and ZY in terms of total FAs and FA compositions (Supplementary Figs S3, S5; Supplementary Tables S17, S19). JR is a low SOC cultivar, whereas ZY has a relatively high SOC. The total FA difference between these two cultivars varied between 10% and 15% when they were grown under local open field conditions, but the total FA difference decreased to only ~5% when they were grown in an enclosed growth chamber. Overall, in comparison with the open field, the enclosed growth chamber caused a more remarkable decrease in total FAs in the high SOC cultivar ZY than in the low SOC cultivar JR. This finding was consistent with the results in Table 4, where two rate-limiting enzymes involved in glyoxylate metabolism, namely isocitrate lyase (ICL) and malate synthase (MLS), were remarkably up-regulated in ZY by high NT. However, only one rate-limiting enzyme, ICL, was up-regulated by high NT in JR, which suggested a high rate of lipid decomposition in ZY.

Two time points to harvest seed samples were designed (Table 1; Fig. 1). The time point 05.00 h in darkness ensured the maximum effect of NT difference. The time point 14.00 h in the daytime was several hours after DT restoration when photosynthesis efficiency should be the highest. The net lipid storage of a matured seed resulted from the amount of synthesized lipids minus the amount of catabolized lipids. SFARs, a group of GDSL lipases, are active in degrading seed FAs at the late stage of seed development (Chen *et al.*, 2012*a*).

Overall, high NT resulted in the reduction of individual FA species and the total FAs in seeds (Fig. 3A). Nonetheless, the significant reduction of some FA species and the non-significant decrease of others altered the proportions of FAs in seeds. In terms of percentage, C18:0 and C18:1 decreased, but C18:2 and C18:3 increased in both cultivars; EA increased in JR, but remained unchanged in ZY (Fig. 3B). Linoleic acid (C18:2) and linolenic acid (C18:3), similarly to other PUFAs, are not easily synthesized in the human body (Parker *et al.*, 2006). They are beneficial to human health due to their functions, such as anticardiovascular, anticerebrovascular, and regulation of the human immune system. In addition, EA (C22:1) is a considerably long-chain FA, which is unhealthy to the human cardiovascular system (Wang *et al.*, 2006). Many studies showed the adaptation of lipid metabolism responding to temperature changes. Nevertheless, the majority of these investigations focused on the alteration of the membrane lipid profile in vegetative organs, such as leaves, under stressful temperature and the role of PUFAs in determining membrane fluidity (Murata and Los, 1997; Murakami *et al.*, 2000; Li *et al.*, 2015). In contrast, the effect of temperature, in particular NT, on seed total FAs and FA compositions has not not widely investigated. A warm climate during the pod filling stage results in SOC reduction in oilseed rape (Zhu *et al.*, 2012; Singer *et al.*, 2016). Investigations on various oil crops provided contradictory results regarding the influence of high temperature on FA composition, especially on C18-FA proportions (Wolf *et al.*, 1982; Dornbos and Mullen, 1992; Piper and Boote, 1999). To our knowledge, all these studies concerned the influence of overall DT and NT. Only a few studies focused solely on the effect of NT on the storage lipid metabolism of oilseeds. In the present study, we showed that high NT up-regulated a variety of lipases degrading storage lipids and the FA desaturating genes (Table 2; Fig. 7). Consequently, the seed proportion of C18:2 and C18:3 increased. Heat stress is negatively correlated with the degree of unsaturation in plant vegetative organs; that is, high growth temperatures result in low PUFAs in leaves (Eastmond and Graham, 2001; Martinière *et al.*, 2011). However, other abiotic stresses, such as drought and cold stresses, increase the proportion of PUFAs (C18:2 and C18:3) because unsaturated lipid substrates are required for membrane desaturation when plants are subjected to stresses (Zhang *et al.*, 2012).

Carbon fixation and starch/sucrose metabolism are fundamental biochemical reactions upstream of lipid metabolism; these reactions provide a precursor substance for TAG synthesis. Our result demonstrated a positive effect of high NT on the expression of genes of carbon fixation and starch/sucrose metabolism in samples harvested at 05.00 h, which was in contrast to the negative effect in samples harvested at 14.00 h (Table 2). High NT could result in remarkable lipid degradation during the night-time, which could consequently stimulate the lipid biosynthesis process. The increased lipid synthesis might require many carbon fixation and starch/sucrose metabolism reactions.

Notably, a considerable number of DEGs of plant hormone signaling pathways involved GA signaling (Table 2; Supplementary Tables S16–S19). However, the differences in GA levels in samples harvested at 05.00 h between high and low NT treatments were not verified. The high GA signal caused by high NT could possibly arise from the weakened DELLA function, not from increased GA synthesis. Our result was consistent with those of previous studies reporting that cool temperature, especially cold stress, strengthens the DELLA function (Eremina *et al.*, 2016). In contrast, warm temperature, not heat stress, weakens DELLAs (Oh *et al.*, 2007; Ozga *et al.*, 2017). GA signal regulates many aspects of plant growth and development, including seed germination, leaf expansion, stem elongation, and development of trichome, flower, and fruit (Cheng *et al.*, 2004; Yamauchi *et al.*, 2007). The C19-GAs, such as GA_1_, GA_3_, GA_4_, and GA_7_ are biologically active, and have intrinsic or inherent growth-promoting activity. Of these, GA_1_ and its precursors are the principal GAs in developing seeds of oilseed rape (*B. napus*) (Rood *et al.*, 1989; Zanewich and Rood, 1993). Recently, GA signal was discovered to up-regulate a group of GDSL-type lipase genes, namely *SFAR* genes, to reduce seed storage lipid in Arabidopsis (Chen *et al.*, 2012*a*). In the present experiment, we observed that GA signal enhancement was accompanied by the up-regulation of *BnSFAR* orthologs (Supplementary Tables S17, S19), with the active involvement of genes in β-oxidation, glyoxylate metabolism, glycolysis/gluconeogenesis, and citrate cycle/tricarboxylic acid cycle pathways (Table 2), possibly due to lipid catabolism. We also observed that genes involved in glyoxylate metabolism pathways noticeably featured a *cis*-acting site GAGA-similar motif at ~1 kb upstream of the start codon (Supplementary Table S20). In *Drosophila*, this motif is conserved in genes encoding heat shock protein (Georgel, 2005).

Various strategies are practiced to increase the oil production of oilseed rape. In this study, we suggested an alternative strategy to increase SOC by inhibiting lipid catabolism via applying paclobutrazol (PAC) to block GA synthesis. We proposed that the net lipid storage of a matured seed resulted from the amount of synthesized lipids minus the amount of catabolized lipids. The balance between lipid anabolism and catabolism could be influenced by a range of external or internal environmental factors. Repressing upstream signaling, such as GA signaling, would be favorable for the net seed storage lipid accumulation by preventing the lipid from being catabolized. In a 2-year and two-location field experiment, we applied PAC to oilseed rape pods approaching maturation. The field experiment result supported the main conclusion that GA signal decreases the seed storage lipid accumulation during seed maturation (Supplementary Fig. S5).

As shown in Fig. 7, the high NT up-regulated GA signaling, thereby causing the active involvement of lipid-degrading enzymes, such as BnSFARs. The free FAs released from TAGs were subject to decomposition in β-oxidation and glyoxylate metabolism pathways, as evidenced by the increased expression of the genes in the two pathways, such as the rate-limiting enzymes MLS and ICL. Thus, high NT reduced the total FAs in both low and high SOC cultivars, namely JR and ZY, and increased the unsaturated C18-FA (C18:2 and C18:3) proportion in both seeds. Spraying PAC on oilseed rape plants at the pod filling stage is proposed to overcome the negative effect of high NT in lowering seed storage lipids.

## Supplementary data

Supplementary data are available at *JXB* online.

Fig. S1. Box plot showing the gene expression level normalized with RPKM (log_10_^X^).

Fig. S2. Top 20 pathways for differentially expressed genes (DEGs) between sample A and C by numbers and rich factors.

Fig. S3. Top 20 pathways for DEGs between sample B and D by numbers and rich factors.

Fig. S4. Top 20 pathways for DEGs between sample E and G by numbers and rich factors.

Fig. S5. Top 20 pathways for DEGs between sample F and H by numbers and rich factors.

Fig. S6. Effect of gibberellin (GA) on the oil yield of oilseed rape (*Brassica napus* L.). (A) Comparison of oil yield between the application of GA, water control, and paclobutrazol (PAC) over 2 years, two locations, and two genotypes (JR and ZY). (B) Comparison of oil yield between the application of GA, water control, and PAC in a particular location, year, and genotype.

Table S1. Gene numbers and read coverages of samples defined in Table 1 mapped against the reference genome of oilseed rape.

Table S2. Gene IDs in SBC1 and their expressional differences among treatments A–H, as shown in Fig. 4.

Table S3. Gene IDs in SBC2 and their expressional differences among treatments A–H, as shown in Fig. 4.

Table S4. Gene IDs in SBC3 and their expressional differences among treatments A–H, as shown in Fig. 4.

Table S5. Gene IDs in SBC4 and their expressional differences among treatments A–H, as shown in Fig. 4.

Table S6. Gene IDs in SBC5 and their expressional differences among treatments A–H, as shown in Fig. 4.

Table S7. Gene IDs in SBC6 and their expressional differences among treatments A–H, as shown in Fig. 4.

Table S8. IDs of DEGs in comparison between treatments A and C.

Table S9. IDs of DEGs in comparison between treatments B and D.

Table S10. IDs of DEGs in comparison between treatments E and G.

Table S11. IDs of DEGs in comparison between treatments F and H.

Table S12. IDs of the enriched DEGs in the top 20 pathways treated with A and B, as visually illustrated in Supplementary Fig. S2.

Table S13. IDs of the enriched DEGs in the top 20 pathways in comparison between treatments C and D, as visually illustrated in Supplementary Fig. S3.

Table S14. IDs of the enriched DEGs in the top 20 pathways in comparison between treatments E and G as visually illustrated in Supplementary Fig. S4.

Table S15. IDs of the enriched DEGs in the top 20 pathways in comparison between treatments F and H, as visually illustrated in Supplementary Fig. S5.

Table S16. IDs of DEGs in pathways involved in or related to fatty acid (FA) metabolism, as listed in the column A/C of Table 2.

Table S17. IDs of DEGs on pathways involved in or relating to FA metabolism, as listed in the column B/D of Table 2.

Table S18. IDs of DEGs in pathways involved in or related to FA metabolism, as listed in the column E/G of Table 2.

Table S19. IDs of DEGs in pathways involved or related to FA metabolism, as listed in the column F/H of Table 2.

Table S20. IDs of genes in the glyoxylate metabolism pathway that have a *cis*-acting site GAGA-similar motif at 1 kb upstream of the start codon of these genes.

Table S21. Primer sequences used for RT-qPCR analysis.

Supplementary Figures S1-S6

Supporting Information Data S1-S21
