# Supplementary material for: Effect of high night temperature on storage lipids and transcriptome changes in developing seeds of oilseed rape
Source: J Exp Bot. 2018 Feb 6;69(7):1721–33. doi: 10.1093/jxb/ery004 (PMC5888911; doi:10.1093/jxb/ery004)
Supplement: Supplementary Figures S1-S6 [file ery004_suppl_supplementary_figures_s1-s6.pdf]

Effect of high night temperature on storage lipids and transcriptome changes in developing seeds of oilseed rape (*Brassica napus* L.)

Longhua Zhou[1,†], Tao Yan[1], Xin Chen[1], Zhilan Li[1], Dezhi Wu[1], Shuijin Hua[2], Lixi Jiang[1,*]

1. Institute of Crop Science, Zhejiang University, Yu-Hang-Tang Road 866, Hangzhou 310058, China
2. Institute of Crop and Nuclear Technology Utilization, Zhejiang Academy of Agricultural Sciences, Shiqiao Road 198, Hangzhou 310021,China

- Author of correspondence: Lixi Jiang, Tel +86 571 8898 2905; Fax +86 571 88982905; Email: jianglx@zju.edu.cn

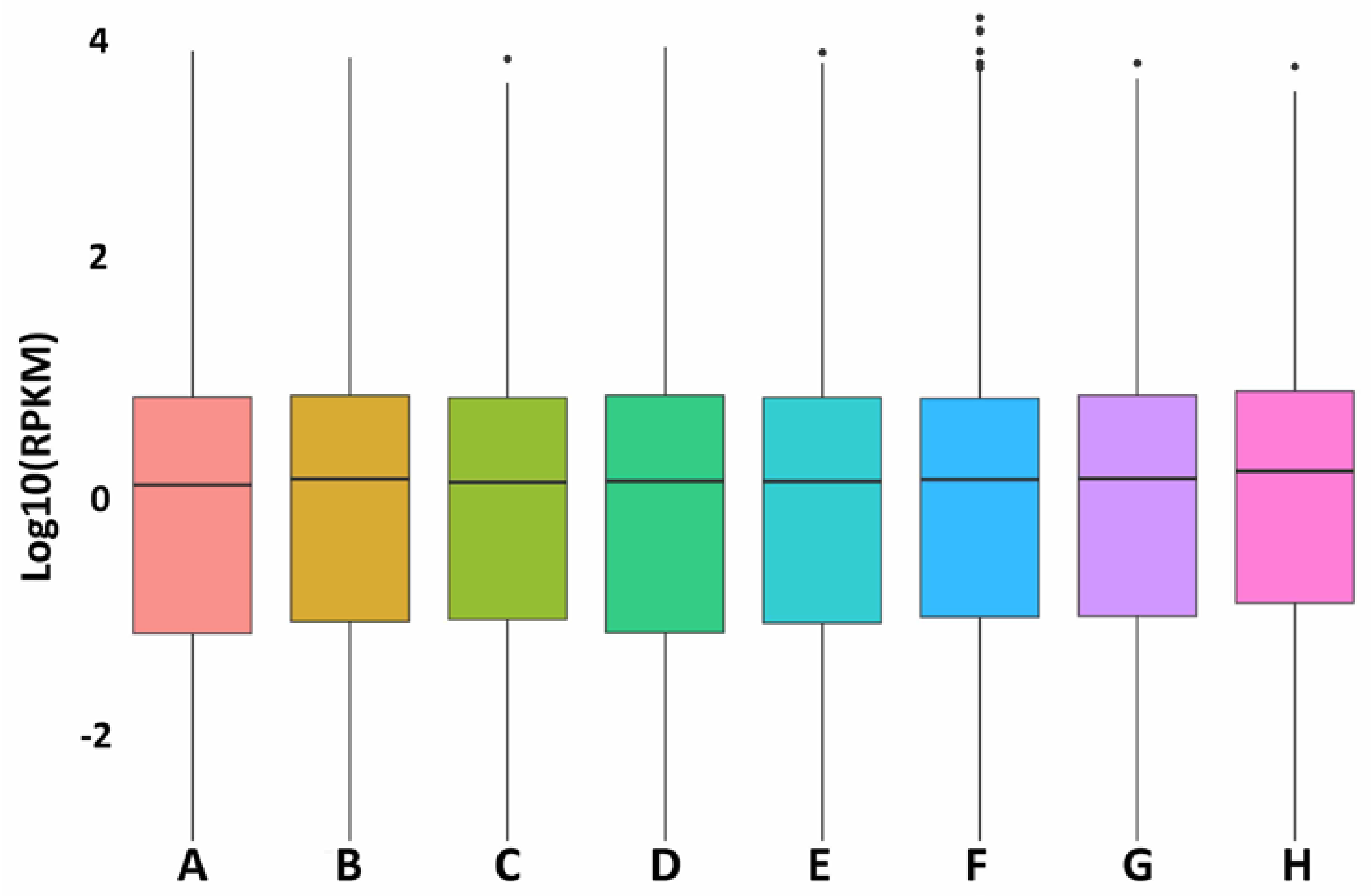

Fig. S1. Box plot showing the gene expression level normalized with reads per kilobase million (Log_{10}^{X}). Samples A–H in different colors represent the eight treatments defined in Table 1. Data are means of two biological repetitions.

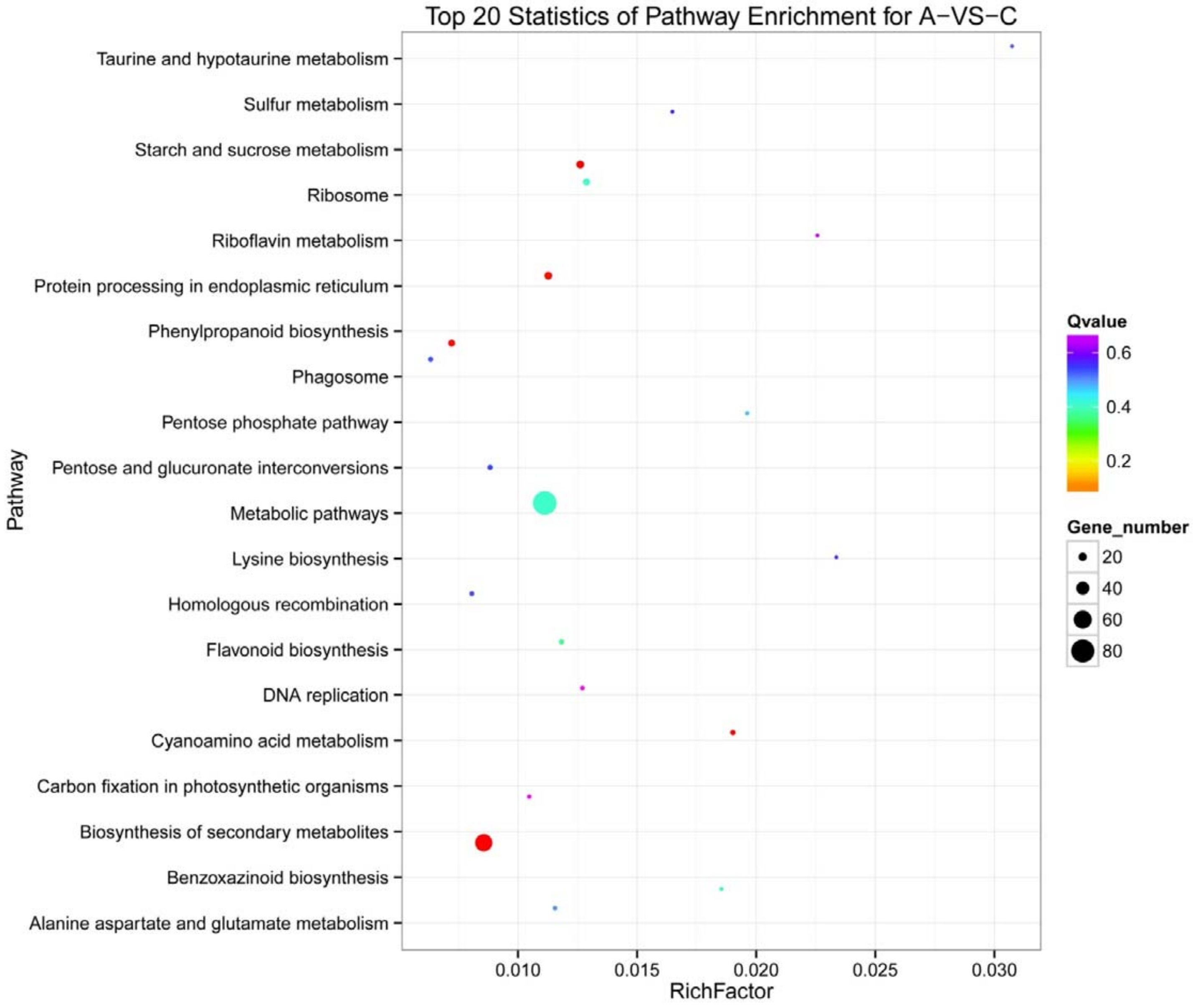

Fig. S2. Top 20 pathways for differentially expressed genes (DEGs) between Sample A and C by numbers and rich factors. Dot sizes from small to large correspond to the increasing number of DEGs. Colors indicate the *Q*-values of Kyoto Encyclopedia of Genes and Genomes (KEGG) analysis.

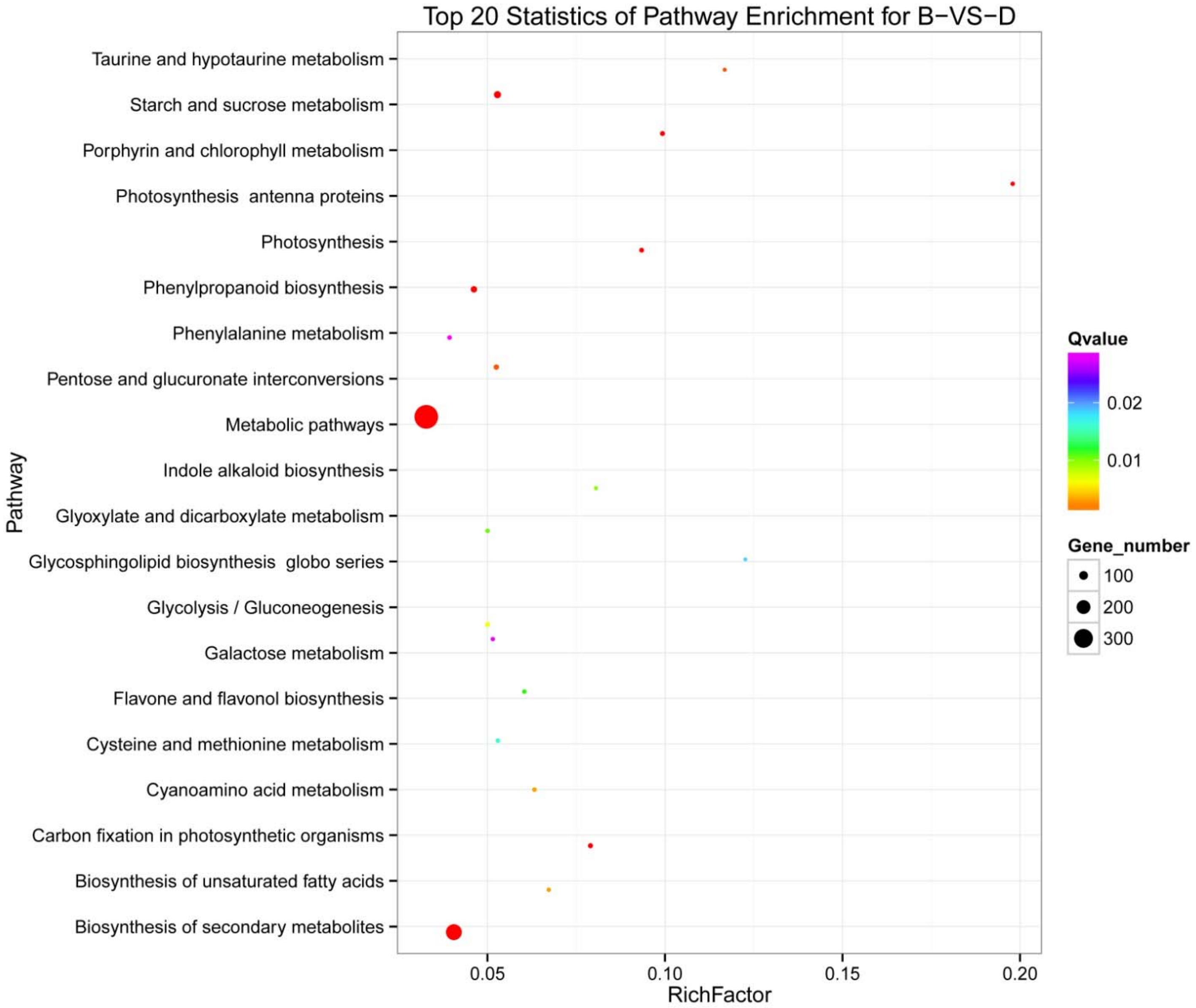

Fig. S3. Top 20 pathways for DEGs between sample B and D by numbers and rich factors. Dot sizes from small to large correspond to the increasing number of DEGs. Colors indicate the Q-values of KEGG analysis.

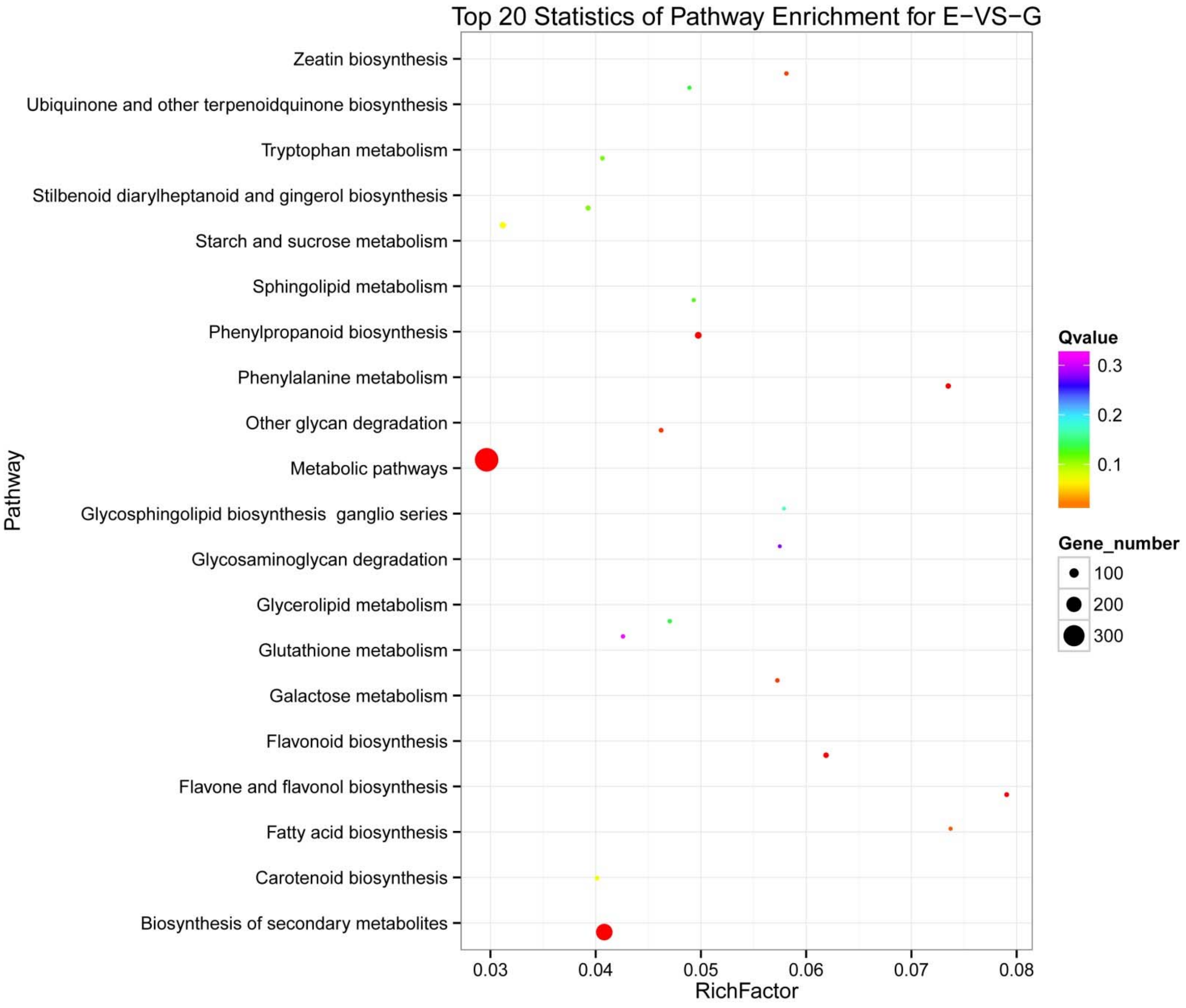

Fig. S4. Top 20 pathways for DEGs between sample E and G by numbers and rich factors. Dot sizes from small to large correspond to the increasing number of DEGs. Colors indicate the Q-values of KEGG analysis.

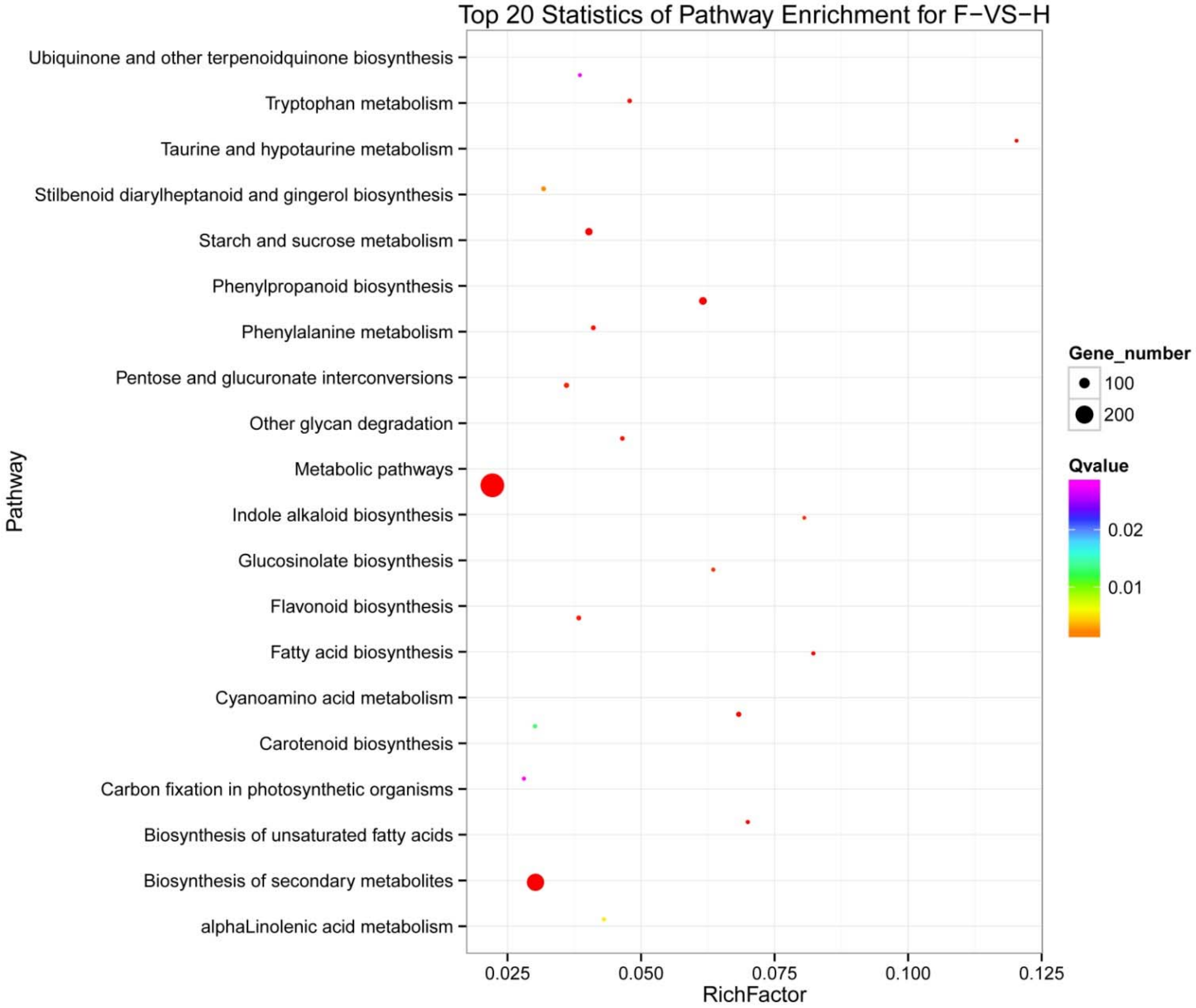

Fig. S5. Top 20 pathways for DEGs between sample F and H by numbers and rich factors. Dot sizes from small to large correspond to increasing number of DEGs. Colors indicate the Q-values of KEGG analysis.

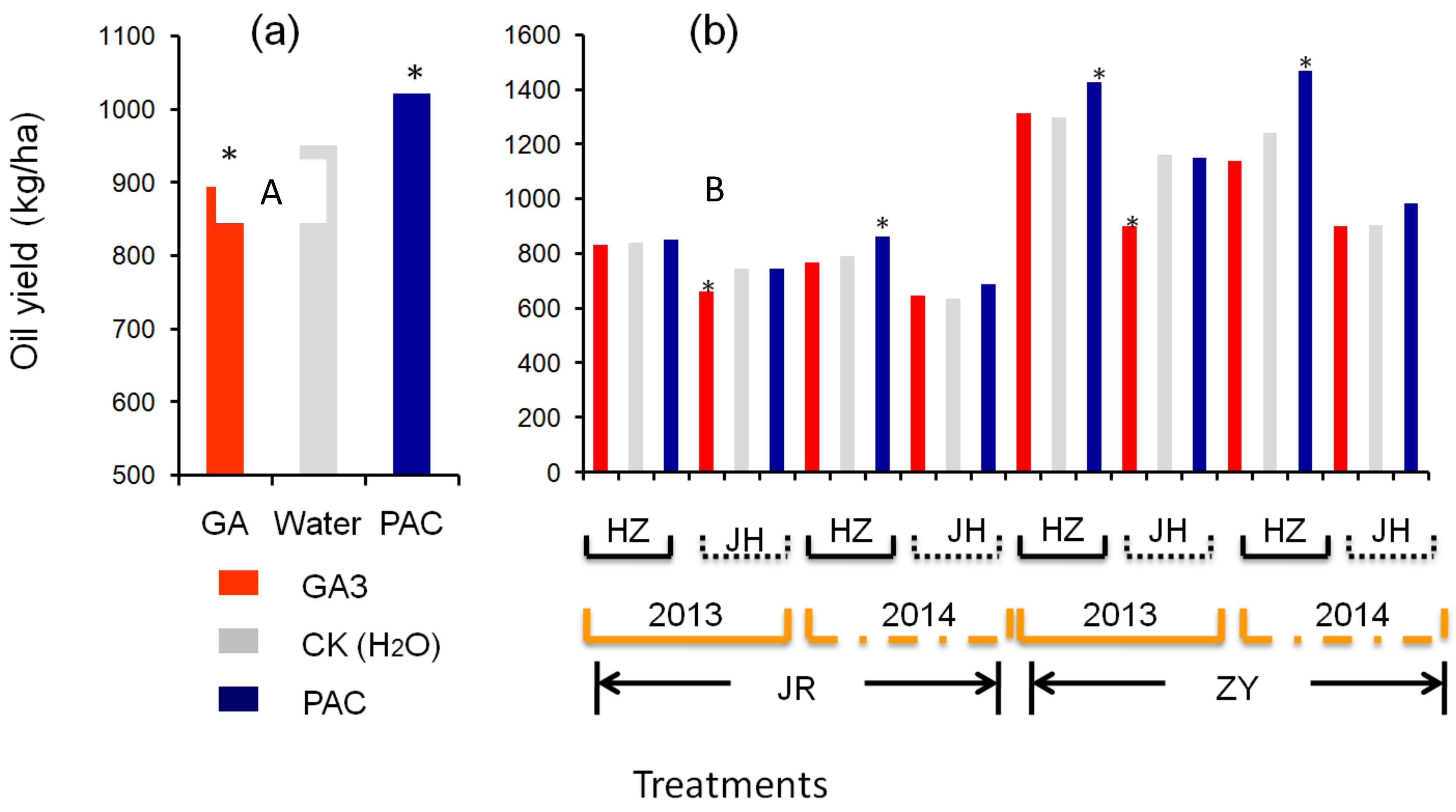

Fig. S6. Effect of gibberellin (GA) on the oil yield of oilseed rape (*Brassica napus* L.). (A) Comparison of oil yield between the application of GA, water control, and paclobutrazol (PAC) over two years, two location, and two genotypes (JR and ZY). (B) Comparison of oil yield between the application of GA, water control, and PAC in particular location, year, and genotype. Asterisks indicate significant differences of GA (or PAC) application from the control. HZ and JH represent Hangzhou and Jinhua locations, respectively. 2013 and 2014 stand for the winter oilseed rape growing seasons in 2012–2013 and 2013–2014, respectively. Asterisks indicate significant difference ($p \leq 0.05$ level) in comparison with the water control.
